# Editorial: Models and Theories of Speech Production

**DOI:** 10.3389/fpsyg.2020.01238

**Published:** 2020-06-19

**Authors:** Adamantios Gafos, Pascal van Lieshout

**Affiliations:** ^1^Department of Linguistics and Excellence Area of Cognitive Sciences, University of Potsdam, Potsdam, Germany; ^2^Department of Speech-Language Pathology, Oral Dynamics Laboratory, University of Toronto, Toronto, ON, Canada

**Keywords:** speech production, motor control, dynamical models, phonology, speech disorders, timing

Spoken language is conveyed via well-coordinated speech movements, which act as coherent units of control referred to as gestures. These gestures and their underlying movements show several distinctive properties in terms of lawful relations among the parameters of duration, relative timing, range of motion, target accuracy, and speed. However, currently, no existing theory successfully accounts for all properties of these movements. Even though models in speech motor control in the last 40 years have consistently taken inspiration from general movement science, some of the comparisons remain ill-informed. For example, our present knowledge on whether widely known principles that apply to limb movements (e.g., the speed-accuracy trade off known as Fitts' law) also hold true for speech movements is still very limited. An understanding of the principles that apply to speech movements is key to defining the somewhat elusive concept of speech motor skill and to assessing and interpreting different levels of that skill in populations with and without diagnosed speech disorders. The latter issue taps into fundamental debates about whether speech pathology assessment paradigms need to be restricted to control regimes that are specific to those underlying typical speech productions. Resolution of such debates crucially relies on our understanding of the nature of speech processes and the underlying control units.

Unlike movements in locomotion or oculomotor function, speech movements when combined into gestures are not mere physical instantiations of organs moving in space and time but, also, have intrinsic symbolic function. Language-particular systems, or phonological grammars, are involved in the patterning of these gestures. Grammar constraints regulate the permissible symbolic combinations as evidenced via eliciting judgments on whether any given sequence is well-formed in any particular language (the same sequence can be acceptable in one, but not the other language). In what ways these constraints shape speech gestures and how these fit with existing general principles of motor control is, also, not clearly understood.

Furthermore, speech gestures are parts of words and thus one window into understanding the nature of the speech production^1^system is to observe speech movements as parts of words or larger chunks of speech such as phrases or sentences. The intention to produce a lexical item involves activating sequences of gestures that are part of the lexical item. The regulation in time of the units in such sequences raises major questions for speech motor control theories (but also for theories of cognition and sequential action in general). Major challenges are met in the inter-dependence among different time scales related to gestural planning, movement execution and coordination within and across domains of individual lexical items. How these different time scales interact and how their interaction affects the observed movement properties is for the most part still unknown.

In this special issue, we present a variety of theoretical and empirical contributions which explore the nature of the dynamics of speech motor control. For practical purposes, we separate these contributions in two major themes:

1) Models and theories of speech production.
2) Applications.

Following is a short description of each paper as listed under these themes.

1) Models and theories of speech production

The speech signal is simultaneously expressed in two information-encoding systems: articulation and acoustics. Goldstein's contribution addresses the relation between representations in these two parallel manifestations of speech while focusing not on static properties but on patterns of change over time (temporal co-modulation) in these two channels. To do so, Goldstein quantifies the relation between rates of change in the parallel acoustic and articulatory representations of the same utterance, produced by various speakers, based on x-ray microbeam data. Analysis of this relation indicates that the two representations are correlated via a pulse-like modulation structure, with local correlations being stronger than global ones. This modulation seems linked to the fundamental unit of the syllable.

It is widely assumed that acoustic parameters for vowels are normally distributed, but it is rarely demonstrated that this might be the case. Whalen and Chen quantified the distributions of F1 and F2 values of /i/ and /o/ in the English words “heed,” “geek,” “ode”/“owed,” and “dote” produced by a single speaker on three different days. Analysis based on a high number of repetitions of these vowels in different consonantal contexts indicates that distributions are generally normal, which in turn suggests consistent vowel-specific targets across different contextual environments. The results add weight to the widely-held assumption that speech targets follow a normal distribution and the authors discuss the implications for theories of speech targets.

Turk and Shattuck-Hufnagel address the nature of timing in speech, with special attention given to movement endpoints, which as they argue relate to the goals of these movements. The argument is presented that these points require dedicated control regimes. Evidence for this argument is derived from work in both speech and non-speech motor control. It is also argued that in contrast to the Articulatory Phonology/Task Dynamics view, where gestural durations are determined by an intrinsic dynamics, duration must be an independently controlled variable in speech. A phonology-extrinsic component is thus proposed to be necessary and a call is made for developing and testing models of speech where a component of abstract, symbolic phonological representations is kept apart from the way(s) in which these representations are implemented in quantitative terms which include surface duration specifications and attendant timing mechanisms for achieving these.

Shaw and Chen investigated to what degree timing between gestures is stable across variations in the spatial positions of individual articulators, as predicted in Articulatory Phonology. Using Electromagnetic Articulography with a group of Mandarin speakers producing CV monosyllables, they found a correlation between the initial position of the tongue gesture for the vowel and C-V timing. In contrast to the original hypothesis, this indicates that inter-gestural timing is sensitive to the position of the articulators, suggesting a critical role for somatosensory feedback.

Roessig and Mücke study tonal and kinematic profiles of different degrees of prominence (unaccented, broad, narrow and contrastive focus) from 27 speakers of German. Parameters in both the tonal and kinematic dimensions are shown to vary systematically across degrees of prominence. A dynamical approach is put forward in modeling these findings. This approach embraces the multidimensionality of prosody while at the same time showing how both discrete and continuous modifications in focus marking can be expressed within one formal language. The model captures qualitatively the observed patterns in the data by tuning of an abstract control variable which shapes the attractor landscape over the parameter space of kinematic and tonal dimensions considered in this work.

Iskarous provides a computational approach to explain the nature of spatiotemporal particulation of the vocal tract, as evidenced in the production of speech gestures. Based on a set of reaction-diffusion equations with simultaneous Turing and Hopf patterns the critical characteristics of speech gestures related to vocal tract constrictions can be replicated in support of the notion that motor processes can be seen as the emergence of low degree of freedom descriptions from high degree of freedom systems.

Patri et al. address individual differences in responses to auditory or somatosensory perturbation in speech production. Two accounts are entertained. The first reduces individual differences to differences in acuity of the sensory specifications while the second leaves sensory specifications intact and, instead, modulates the sensitivity of match between motor commands and their auditory consequences. While simulation results show that both accounts lead to similar results, it is argued that maintaining intact sensory specifications is more flexible, enabling a more encompassing approach to speech variability where cognitive, attentional and other factors can modulate responses to perturbations.

One of the foundational ideas of phonology and phonetics is that produced and perceived utterances are decomposed into sequences of discrete units. However, evidence from development indicates that in child speech utterances are holistic rather than segmented. The contribution by Davis and Redford offers a theoretical demonstration along with attendant modeling that the posited units can emerge from a stage of speech where words or phrases start off as time-aligned motoric and perceptual trajectories. As words are added and repeatedly rehearsed by the learner, motoric trajectories begin to develop recurrent articulatory configurations which, when coupled with their corresponding perceptual representations, give rise to perceptual-motor units claimed to characterize mature speech production.

In their contribution, Kearney et al. present a simplified version of the DIVA model, focusing on three fitting parameters related to auditory feedback control, somatosensory feedback control, and feedforward control. The model is tested through computer simulations that identify optimal model fits to six existing sensorimotor adaptation datasets, showing excellent fits to real data across different types of perturbations and experimental paradigms.

An active area in phonological theory is the investigation of long-distance assimilation where features of a phoneme assimilate to features of another non-adjacent phoneme. Tilsen seeks to identify mechanisms for the emergence of such non-local assimilations in speech planning and production models. Two mechanisms are proposed. The first is one where a gesture is either anticipatorily selected in an earlier epoch or is not suppressed (after being selected) so that its influence extends to later epochs. The second is one where gestures which may be active in one epoch of a planning-level dynamics, even though not selected during execution, may still influence production in a different epoch. Evidence for these mechanisms is found in both speech and non-speech movement preparation paradigms. The existence of these two mechanisms is argued to account for the major dichotomy between assimilation phenomena that have been described as involving the extension of an assimilating property vs. those that cannot be so described.

Xu and Prom-on contrast two principles assumed to underlie the dynamics of movement control: economy of effort and maximum rate of information. They present data from speakers of American English on repetitive syllable sequences who were asked to imitate recordings of the same sequences that had been artificially accelerated and to produce meaningful sentences containing the same syllables at normal and fast speaking rates. The results show that the characteristics of the formant trajectories they analyzed fit best the notion of the maximum rate of information principle.

Kröger et al.'s contribution offers a demonstration that a learning model based on self-organizing maps can serve as bridge between models of the mental lexicon and models of sensorimotor control and that such a model can learn (from semantic, auditory and somatosensory information) representational units akin to phonetic-phonological features. At a broad level, few efforts have been made to bridge theory and modeling of the lexicon and motor control. The proposed model aims at addressing that gap and makes predictions about the specificity and rate of growth of such representational features under different training conditions (auditory only vs. auditory and somatosensory training modes).

Parrell and Lammert develop a synthesis of the dynamic movement primitives model of motor control (Schaal et al., 2007; Ijspeert et al., 2013) with the task dynamics model of speech production (Saltzman and Munhall, 1989). A key element in achieving this synthesis is the incorporation of a learnable forcing term into the task dynamics' point-attractor system. The presence of such a tunable term endows task dynamics with flexibility in movement trajectories. The proposed synthesis also establishes a link to optimization approaches to motor control where the forcing term can be seen to minimize a cost function over the timespan of the movement under consideration (e.g., minimizing total energy expended during a reaching movement). The dynamics of the proposed synthesis model are explicitly described and their effects are demonstrated in the form of proof of concept simulations showing the consequences of perturbations on jaw movement trajectories.

2) Applications

Noiray et al. present a study in which they examined whether phonemic awareness correlates with coarticulation degree, commonly used as a metric for estimating the size of children's production units. A speech production task was designed to test for developmental differences in intra-syllabic coarticulation degree in 41 German children from 4 to 7 years of age, using ultrasound imaging. The results suggest that the process of developing spoken language fluency involves dynamical interactions between cognitive and speech motor domains.

Tiede et al. describe a study in which they tracked movements of the head and speech articulators during an alternating word pair production task driven by an accelerating rate metronome. The results show that as production effort increased, so did speaker head nodding, and that nodding increased abruptly following errors. The strongest entrainment between head and articulators was observed at the fastest rate under coda alternation conditions.

Namasivayam et al. present an Articulatory Phonology approach for understanding the nature of Speech Sound Disorders (SSDs) in children, aiming to reconcile the traditional phonetic-phonology dichotomy with the concept of interconnectedness between these levels. They present evidence supporting the notion of articulatory gestures at the level of speech production and how this is reflected in control processes in the brain. They add an overview of how an articulatory “gesture”-based approach can account for articulatory behaviors in typical and disordered speech production, concluding that the Articulatory Phonology approach offers a productive strategy for further research in this area.

Heyne et al.  address the relation between speech and another oral motor skill, trombone playing. Using ultrasound, they recorded midsagittal tongue shapes from New Zealand English and Tongan-speaking trombone players. Tongue shapes from the two language groups were estimated via fits with generalized additive mixed models, while these speakers/players produced vowels (in their native languages) and sustained notes at different pitches and intensities. The results indicate that, while airflow production and requisite acoustics largely constrain vocal tract configuration during trombone playing, evidence for a secondary influence from speech motor configurations can be discerned in that the two groups tended to use different tongue configurations resembling distinct vocalic monopthongs in their respective languages.

The papers assembled for this Research Topic attest to the advantages of combining theoretical and empirical approaches to the study of speech production. They also attest to the value of formal modeling in addressing long-standing issues in speech development and the relationship between motor control and phonological patterns; to the importance of somatosensory and auditory feedback in planning and monitoring speech production and the importance of integrating speech production models with other aspects of cognition; and finally, to the potential of theoretical models in informing applications of speech production in disordered speech and motor skills in other oral activities such as playing musical instruments.

## Author Contributions

All authors listed have made equal contributions to the work and approved it for publication.

## Conflict of Interest

The authors declare that the research was conducted in the absence of any commercial or financial relationships that could be construed as a potential conflict of interest.

## Footnotes

^1^One of our reviewers notes that in the field of psycholinguistics the term speech production is used more broadly (than in the use of the term implied by the contributions to this Research Topic) and, points out the need, aptly stated, “to bridge the gap between psycholinguistically informed phonetics and phonetically informed psycholinguistics.” We fully concur and look forward to future research efforts and perhaps Research Topics devoted to such bridging. For a recent special issue on psycholinguistic approaches to speech production, see Meyer et al. (2019) and for a more focused review of the issues pertinent to “phonetic encoding” (a term in psycholinguistics roughly equivalent to our use of the term speech production in the present Research Topic) see Laganaro (2019).
